# Association of Endothelial Cell Activation with Acute Kidney Injury during Coronary Angiography and the Influence of Recombinant Human C1 Inhibitor—A Secondary Analysis of a Randomized, Placebo-Controlled, Double-Blind Trial

**DOI:** 10.3390/biomedicines12091956

**Published:** 2024-08-27

**Authors:** Stephan Moser, Laura Araschmid, Anneza Panagiotou, Leo H. Bonati, Tobias Breidthardt, Gregor Fahrni, Christoph Kaiser, Raban Jeger, Marten Trendelenburg, Michael Osthoff

**Affiliations:** 1 Division of Internal Medicine, University Hospital Basel, 4031 Basel, Switzerland; 2 Department of Clinical Research, University of Basel, 4001 Basel, Switzerland; 3 Research Department, Reha Rheinfelden, 4310 Rheinfelden, Switzerland; 4 Department of Cardiology, Stadtspital Triemli, 8063 Zürich, Switzerland; 5 Department of Cardiology, University Hospital Basel, 4031 Basel, Switzerland; 6 Division of General Internal Medicine, Cantonal Hospital Winterthur, 8400 Winterthur, Switzerland

**Keywords:** endothelial cell activation, complement system, C1 inhibitor, contrast media, ICAM-1, VCAM-1, E-selectin, CCL5

## Abstract

Background: Acute kidney injury (AKI) as a result of iodinated contrast media (CM) has been linked to CM-induced renal ischemia and toxic effects on endothelial cells (EC). The recombinant human C1 inhibitor (rhC1INH) has been shown to influence EC activation. Methods: Secondary analysis of 74/77 (96%) participants of a double-blind, randomized, and placebo-controlled study that assessed the effect of rhC1INH on AKI. E-selectin, intercellular adhesion molecule-1 (ICAM-1), vascular cell adhesion molecule (VCAM-1), and CC-chemokin-ligand-5 (CCL5) were determined in frozen blood samples over 48 h and analyzed according to the treatment group and renal outcomes. Results: The mean age was 76.7 years, and 37 patients each received rhC1INH and placebo, respectively. In the entire study population, minor differences in median EC activation markers/CCL5 concentrations during the first 48 h compared to baseline were observed (e.g., E-selectin 27.5 ng/mL at baseline vs. 29.7 ng/mL on day 1, CCL5: 17.7 ng/mL at baseline vs. 32.2 ng/mL on day 2). Absolute changes in ICAM-1/E-selectin concentrations correlated with a higher peak change in urinary NGAL concentrations. However, AKI was not associated with significant changes in EC markers/CCL5. Last, no significant differences in serum concentrations of EC activation markers/CCL5 were evident between the placebo and the rhC1INH group. Conclusions: CM administration during coronary angiography only mildly activated ECs within the first 48 h, which does not explain subsequent AKI. The administration of rhC1INH was not associated with a reduction of EC activation or CCL5.

## 1. Introduction

Iodinated contrast agents used in diagnostic imaging and intravascular therapeutic interventions have been linked to contrast-associated acute kidney injury (CA-AKI), with a consecutive decline in renal function [1]. The exact pathophysiological mechanism of CA-AKI is not yet fully understood, but several indirect and direct effects have been suggested such as direct renal tubular and endothelial cell (EC) cytotoxicity (e.g., by reactive oxygen species) and renal ischemia/reperfusion (I/R) injury as a result of sustained renal vasoconstriction in response to CM [2]. Previously, it was reported as the third most common reason for in-hospital renal failure, recent data indicate a much lower incidence [3].

ECs, in their function as a physical barrier between the intra- and the extravascular compartments, are essential for maintaining body homeostasis and integrity [4,5,6]. EC activation, e.g., in response to pro-inflammatory cytokines such as interleukin-1 (IL-1) or tumor necrosis factor-α (TNF-α), leads to upregulation of cell adhesion molecules such as E-selectin, vascular cell adhesion molecule 1 (VCAM-1), or intercellular cell adhesion molecule 1 (ICAM-1) [7,8], as well as secretion of inflammatory mediators and cytokines such as CC-chemokin-ligand-5 (CCL5) with chemotactic effects [9]. ICAM-1 and VCAM-1 are part of the immunoglobulin superfamily and promote the adhesion of leucocytes and diapedesis [7]. Alternative splicing and proteolytic cleavage are responsible for the soluble form of ICAM-1 (sICAM-1) and VCAM-1 (sVCAM-1) [10,11]. E-selectin on the other hand is expressed by EC at the site of active inflammation and facilitates leucocyte rolling along the endothelial layer before leucocyte adhesion takes place [12]. Although differences in the time- and dose-dependent responses of the various markers to EC activation have been discussed [13], soluble forms of ICAM-1, VCAM-1, E-selectin, and cytokines such as CCL5 are commonly used as biomarkers to measure EC activation [7].

The complement system (CS) is an ancient host defense system and consists of more than 30 serum proteins that are organized in cascades and are activated by the protease activity of the antecedent protein. The cascade is initiated by three pathways: the classical-, the lectin-, and the alternative pathway, which converge later to form the membrane attack complex (MAC) [14], but also lead to opsonization by C3b, activate the inflammatory cascade via the anaphylatoxins C3a and C5a and are involved in the processes of coagulation and fibrinolysis [15]. The CS interacts with EC in various ways. The ECs act thereby as a source, barrier, and target of CS activity [16]. For example, exposure of ECs to serum cytokines such as IFN-γ, IL-1, TNF-α, or IL-6 leads to upregulation of C3 expression. On the other hand, activation of ECs by CS components such as C5a or C5b-9 triggers the expression of adhesion molecules, production of cytokines, chemokines, and growth factors, as well as the propagation of a prothrombotic state [17]. Uncontrolled CS activity or deficiency may violate endothelial integrity, resulting in diseases such as hereditary angioedema, systematic lupus erythematosus, atypical hemolytic syndrome, or age-related macular degeneration [18]. EC heterogeneity, which enables the fulfillment of the diverse vascular functions of the regional and organ-specific vascular segments, plays a decisive role with regard to the specific underlying pathophysiologies [16]. 

C1 inhibitor (C1INH) is a plasma protein that interacts with ECs in various ways, e.g., by binding to E-selectins inhibiting leukocyte-endothelial adhesion and has multiple biological functions and targets, including potent inhibition of the CS and kallikrein-kinin system [19]. Conestat alfa is a recombinant human C1INH (rhC1INH) approved for the substitution treatment of hereditary angioedema. In experimental murine models of acute renal ischemia, administration of rhC1INH was associated with a significant reduction in tubular injury and apoptosis, a decrease in complement deposition, reduced renal infiltration of inflammatory cells, diminished fibrosis, and improved renal function [20,21]. Few data are available in humans, but a previous randomized controlled trial demonstrated that administration of rhC1INH prior to the use of CM in high-risk patients undergoing elective coronary angiography may prevent renal damage [22].

Given the potential involvement of the complement system during angiography after administration of CM, the interaction of the complement system with ECs, and the influence of C1INH on EC activation the aim of this study was to investigate the course of EC activation markers in the setting of elective coronary angiography with associated exposure to CM, their association with renal outcomes, as well as the effect of rhC1INH on EC activation.

## 2. Materials and Methods

### 2.1. Study Description

For the present post hoc analysis, frozen stored blood samples from the prophylactic RhC1-inhibitor to prevent contrast-induced nephropathy (PROTECT) study were used. The PROTECT trial, an investigator-initiated, single-center, randomized, double-blind, placebo-controlled, phase II clinical trial, investigated the prophylactic effect of rhC1INH in patients with chronic kidney disease undergoing elective coronary angiography [22]. This study was conducted according to the principles of the Declaration of Helsinki and approved by the local ethics committee (EKNZ identifier 2016-01112). Written informed consent was obtained from all study participants. The trial was registered at www.clinicaltrials.gov (NCTO2869347). 

Patients undergoing elective coronary angiography were recruited from January 2017 to May 2018 at the University Hospital of Basel, Switzerland, a 700-bed academic tertiary care center. Inclusion criteria were estimated glomerular filtration rate (eGFR) of ≤50 mL/min/1.73 m^2^ (as calculated by the Chronic Kidney Disease Epidemiology Collaboration study equation) and at least one of the following risk factors for CA-AKI: diabetes mellitus, age ≥75 years, anemia (baseline hematocrit value ≤39% for men and ≤36% for women), congestive heart failure functional class III or IV by New York Heart Association classification, or a history of pulmonary edema. Important exclusion criteria included contraindications to the class of drugs under study, a history of allergy to rabbits, decompensated heart failure or myocardial infarction in the previous 2 weeks, exposure to iodinated CM in the previous 7 days, and dialysis. Included patients were randomized in a 1:1 ratio stratified by the planned procedure (angiography as work-up before transcatheter aortic valve replacement or angiography and potentially angioplasty). Patients in the intervention group received intravenous treatment with rhC1INH (Conestat alfa, 50/U kg bodyweight with a maximum of 4200 U) immediately before and 4 h after the angiography, whereas patients in the placebo group received intravenous 0.9% sodium chloride in an equal volume.

Frozen (−74 °C) blood and urine samples collected at different time points (baseline, after the second administration of the study drug (4 h after angiography), and on day 1 and day 2) were used for the present study. The primary efficacy outcome of the original trial was the peak change in neutrophil gelatinase-associated lipocalin (NGAL), a surrogate marker for kidney injury, in urine within 48 h after coronary angiography. Secondary efficacy outcomes included the development of CA-AKI (defined as serum creatinine increase of >0.3 mg/dL or >50%) within 48 h and the occurrence of an increase in serum cystatin C ≥ 10% within 24 h.

### 2.2. Outcomes

For the present study, the following endpoints were defined: Comparison of the absolute and relative changes in the above-mentioned EC markers (ICAM-1, VCAM-1, E-selectin) and CCL5 in the 48 h post-intervention period in the overall population as well as in subgroups such as patients undergoing percutaneous coronary intervention (PCI) or receiving antiplatelet or anticoagulation treatment. Associations of the EC markers with renal injury markers and renal outcome as defined in the original trial were also investigated. Last, the impact of rhC1INH treatment compared to placebo on the course of the EC activation markers was investigated.

### 2.3. Laboratory Assessment

ICAM-1, VCAM-1, E-selectin, and CCL5 concentrations in stored serum samples were determined using a multiplexed enzyme-linked immunosorbent assay according to the manufacturer’s introduction (ProteinSimple, Bio-Techne, Minneapolis, MN, USA). For the measurements, serum samples were thawed and immediately analyzed with the automated assay using a sample dilution of 1:100.

### 2.4. Statistical Analysis

Continuous, not normally distributed variables were reported as median (interquartile range) and compared using nonparametric tests (Mann–Whitney U for unpaired- and Friedmann–ANOVA/Wilcoxon tests for paired observations). If normally distributed, continuous variables were reported as mean ± standard deviation (SD) and compared via dependent or independent *t*-tests. Categorical variables were expressed as proportions and counts (n (%)) and compared using the chi-square test and cross tables. To investigate relationships between not-normally distributed continuous variables, Spearman correlation tests were conducted. Tests were done at the 2-sided 5% significance level. In the case of multiple comparisons, a Bonferroni correction was applied. A power calculation for the primary endpoint of the original trial was not performed because of a lack of suitable data on preventive therapy studies of C1INH. In analogy to previous interventional studies using different prophylactic regimens and similar outcomes, it was calculated that 40 subjects in each arm are required to allow for the detection in the primary endpoint of 100 ng/mL (power 80%, 2-sided type 1 error of 5%). All analyses were performed with the use of SPSS version 22 software (IBM, Chicago, IL, USA).

## 3. Results

### 3.1. Baseline Characteristics of the Study Population

The current analysis included blood samples from 37 patients in each of the rhC1INH and placebo groups (74/77 (96%) of the enrolled patients in the original trial). 

The mean age (SD) of the entire study population was 76.7 (±8.1) years, and 52 (70.3%) of the patients were men. The percentage of patients suffering from coronary artery disease (56.8%) and peripheral artery disease (13.5%) or risk factors for atherosclerotic diseases such as dyslipidemia (63.5%), smoking (59.5%), and diabetes mellitus (40.5%) was high. Baseline characteristics in the placebo and rhC1INH groups were equally distributed including the percentage of procedural interventions (PCI, 38% in both the placebo and the intervention group). The demographic and clinical characteristics are shown in Table 1.

### 3.2. Serum Concentrations of Endothelial Cell Activation Markers/CCL5 in the Overall Study Population

**Figure 1:**
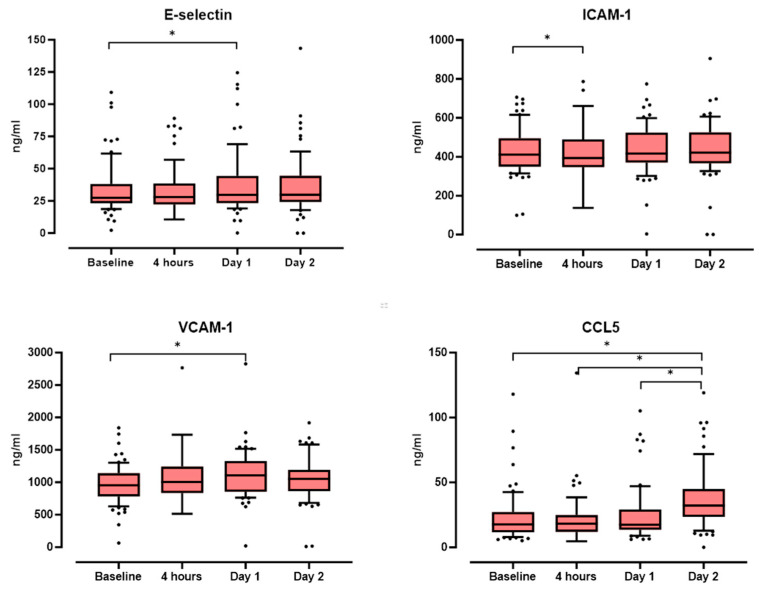
Serum concentrations of CCL5, E-Selectin, ICAM-1, and VCAM-1 (ng/mL) in the overall study population (n = 74) over time. Asterisks denote statistical significance (*p* < 0.05).

In the overall study population, median serum concentrations of all EC markers/CCL5 changed significantly over the observation period of 48 h (E-selectin (Friedmann-ANOVA: *p* = 0.003), ICAM-1 (*p* < 0.001), VCAM-1 (*p* < 0.001) and CCL5 (*p* < 0.001)). The post hoc comparison revealed for E-selectin a significant increase in the median serum concentration from 27.5 ng/mL (IQR 23.2–38.2) at baseline to 29.7 ng/mL (IQR 23.3–44.4) at day 1 (*p* < 0.001) as well as for VCAM-1 (median 954.7 ng/mL (IQR 782.8–1140.7) at baseline to median 1107.2 ng/mL (IQR 854.5–1329.7) on day 1, *p* < 0.001) (Figure 1, Supplementary Table S1). For ICAM-1, there was a significant decrease in median serum concentration from baseline 411.0 ng/mL (IQR 349.2–495.3) to 393.6 ng/mL (IQR 346.6–489.1) at 4 h (*p* = 0.033). However, the most striking difference was demonstrated for CCL5 with a significant increase on day 2. 

Regarding the relative changes E-selectin, ICAM-1, and VCAM-1 showed minor changes over 48 h compared to baseline. Only CCL5 increased by 21.5% (IQR −18.7–70.9) after 4 h and 91.2% (IQR 13.5–178.6) after 48 h. 

**Figure 2:**
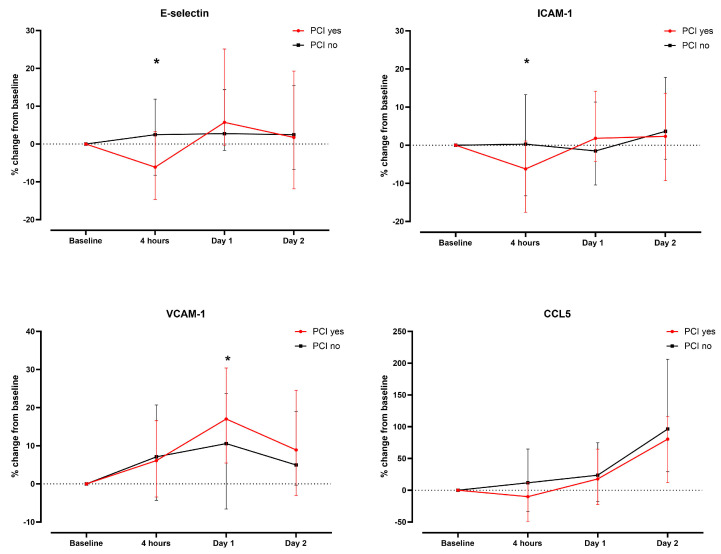
Comparison of relative changes in serum concentrations of endothelial activation markers/CCL5 in patients according to the treatment with percutaneous coronary intervention (PCI) vs. coronary angiography only. Asterisks denote statistical significance (*p* < 0.05).

In the subgroup of patients who underwent PCI, higher median E-selectin serum concentrations on day 1 and day 2 were observed compared to patients without PCI, which was not significant after correction for multiple testing. There was no difference regarding absolute serum concentration in any other EC biomarker assessed (Supplementary Table S2). Concerning the relative change in serum concentrations, a significant difference between groups was found from baseline to 4 h for E-selectin and ICAM-1, and for VCAM-1 on day 1 (Figure 2).

Similarly, there were no significant differences in serum concentrations in patients with or without antiplatelet treatment after correction for multiple tests. Comparing the relative change in serum concentration, only for ICAM-1 a significant difference was seen between baseline vs. 4 h (*p* = 0.037), if correction for multiple comparisons was applied (Supplementary Table S3, Supplementary Figure S1). 

When comparing the subpopulations with and without anticoagulation, significantly different EC concentrations were only found for VCAM-1 at baseline after correction for multiple testing (median 1132 ng/mL (IQR 1034–1427) vs. 949 (IQR 842–1115), *p* = 0.005) (Supplementary Table S4).

Lastly, when comparing patients with chronic kidney disease stage 3 vs. stages 4/5, there was no difference in serum concentrations or increase over time for all endothelial activation markers assessed. 

### 3.3. Association of Endothelial Activation Marker/CCL5 with Renal Injury Markers and Renal Outcome

At baseline, EC activation marker/CCL5 did not correlate with either serum creatinine, cystatin C, or eGFR with the exception of higher baseline VCAM-1 concentration in patients with higher baseline cystatin C concentrations (r = 0.39, *p* = 0.001). Absolute peak change in urinary NGAL within 48 h modestly correlated with only ICAM-1 concentration at baseline (r = 0.41, *p* = 0.001). This was also true for absolute changes in ICAM-1 and E-selectin concentrations compared to baseline indicating a higher absolute increase in patients with a higher peak change in urinary NGAL. However, the correlation was weak (e.g., from r = 0.26, *p* = 0.023 for the correlation with the ICAM-1 increase at 4 h to r = 0.35, *p* = 0.004 for the correlation with the E-selectin increase at day 2).

Regarding renal outcomes, an increase in cystatin C of ≥10% within 24 h and CA-AKI was observed in 18 patients (24.3%) and 12 patients (16%, only stage 1 according to KDIGO criteria), respectively. Serum levels of EC markers/CCL5 and their relative increase compared to baseline were not different in patients with or without the occurrence of the cystatin C or creatinine-based outcome (Table 2. Furthermore, there was no significant correlation of EC activation markers/CCL5 or their changes over 48 h with an increase in serum creatinine during 48 h or the amount of CM administered during coronary angiography.

### 3.4. Association of Treatment with rhC1INH with Endothelial Activation Markers and CCL5

**Figure 3:**
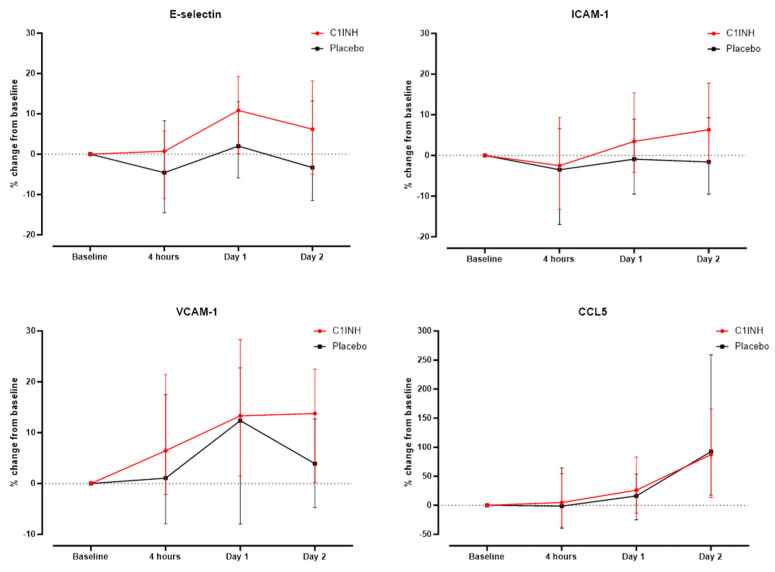
Comparison of relative changes in serum concentrations of endothelial activation markers/CCL5 in patients according to the treatment rhC1INH or placebo.

The absolute concentration of all markers tested was similar in the placebo and rhC1INH groups at all time points (Supplementary Table S5). Similarly, there was no significant difference in the relative changes compared to baseline after correction for multiple testing (Figure 3). 

Analyses of the PCI subgroup of patients according to the intervention yielded results consistent with the overall study population as stated above.

## 4. Discussion

Iodinated CM has been causally linked to acute kidney injury, which is thought to result from CM-induced local renal ischemia and direct toxic effects on ECs. RhC1INH, a multi-target inhibitor of the complement and kinin kallikrein system may interfere with EC activation, which may eventually lead to reduced renal ischemic damage in experimental models. However, in the current study, neither was the occurrence of AKI nor the administration of rhC1INH associated with EC activation after CM administration during coronary angiography. 

Healthy EC function maintains an anti-inflammatory and antithrombotic state which is essential for proper kidney function. Histological observations of a kidney biopsy specimen of patients who suffered from acute tubular necrosis have shown that the de novo synthesis of ICAM-1 and VCAM-1 in the renal tubuli correlates with tissue damage [23]. CA-AKI has been thought to result from CM-mediated IR injury and direct toxic effect on the tubular cells and ECs. Hence, we expected a stronger increase in EC activation markers in patients with CA-AKI. However, we did not observe any differences regardless of the definition of contrast-induced renal injury including serum creatinine or cystatin C. In line, the amount of CM did not correlate with any EC activation marker or course over time. Compared to creatinine, cystatin C is an even more sensitive marker to detect renal damage [24]. The lack of difference may be related to the low incidence of CA-AKI and cystatin C increase of ≥ 10% within 24 h in the study population (less than 20%), and the overall small increase in EC activation markers after angiography which may limit the power of the analysis. 

As kidney injury occurs, the expression of NGAL by tubular epithelial cells is upregulated multiple times [25]. Interestingly, data suggests that NGAL levels are also expressed in patients with cardiovascular diseases [26]. A weakly positive correlation was observed between the absolute urinary NGAL peak increase over 48 h and the absolute change in E-selectin and ICAM-1 from baseline to 4 h, which may not be sufficient to indicate that a common trigger (i.e., contrast media) causes not only tubular damage but also activation of ECs. 

Regarding the entire study population, significant but minor changes in the median serum concentrations of EC activation markers or CCL5 were observed for different time points during the first 48 h after coronary angiography. It seems that CM administration during angiography does not elicit a strong EC response. In vitro models, examining the time course of EC activation after stimulation with interleukin-1β and TNF-α showed contradictory results [27,28]. Li et al. demonstrated an increase in ICAM-1 and VCAM 1 expression after 8 h of stimulation with TNF-α and LPS, whereas Scholz et al. observed the beginning of VCAM-1 and ICAM-1 synthesis after 1 h with a maximum concentration reached after 4–8 h (VCAM-1) and 6–72 h (ICAM-1). Synthesis of E-selectin started after one hour with a maximum after 2.5–4 h. In line, an increase in E-selectin was seen after 4 h. Nonetheless, the relevance of in vitro studies might be questioned, as multiple factors influencing EC activation are not accounted for in experimental models such as treatment with statins or antiplatelet agents. For example, statins are suggested to decrease serum concentrations of ICAM-1, E-selectin, and P-selectin as they improve the inflammatory state of the endothelium via various mechanisms [29]. As 66% patients were on a therapy with statins in our study population, this may explain, among other factors, the lack of more pronounced changes in our study.

Interestingly, the impact of PCI, which may be perceived as a stronger trigger for EC activation, was minor. Only the E-selectin concentrations on day 1 and day 2 differed (not significant after multiple testing), as was the relative change from baseline to 4 h for E-selectin and ICAM-1 and to 1 day for VCAM-1. However, most EC activation marker concentrations were already higher at baseline in the PCI group which may reflect an assumed poorer vascular condition in the PCI group. In contrast to our results, a previous study showed a significant decrease in E-selectin and an increase in VCAM-1 with peak values three days after PCI [30]. On the other hand, Boos et al. saw a significant increase in E-selectin that peaked 24 h after PCI [31]. Of note, all studies, including the present study are limited by the small sample size (n = 36–48 patients). 

Besides its effect on the contact system, coagulation system, and fibrinolytic system, rhC1INH inhibits the classical pathway of the complement system via C1r and C1s and the lectin pathway via MASP1 and MASP2. The complement system and endothelial cell activation are linked via various regulatory mechanisms and rhC1INH is suggested to reduce complement activation and thus endothelial activation. In the present study, we did not observe any significant differences in the serum concentrations of EC markers/ CCL5 between the placebo and the rhC1INH group at any point in time. This was also true for the subgroup of patients undergoing PCI. If at all, a decrease in E-selectin and ICAM-1 was observed in the placebo group, while in the rhC1INH group, these EC activation markers were higher compared to baseline. 

C1INH may interfere with EC activation in several instances including a reduced expression of C3a and C5a, and binding to P- and E-selectin reducing leukocyte-endothelial cell adhesion [32]. However, its lack of significant inhibition of the alternative pathway could be a weak point, as all three pathways of the complement systems converge at the level of C3 and consequently lead to the formation of MAC. Studies have shown that both, MAC and even the cytological inactive form of MAC stimulate ECs. MAC may increase the expression of P- selectin and IL-1, and the inactive form can also bind to ECs and stimulate the expression of ICAM- 1 and VCAM-1 [33]. This leads to profound EC activation, which apparently cannot be attenuated by C1INH, at least not in the chosen dosage. As previously mentioned, our results even showed a trend towards a higher relative increase after 48 h in the rhC1INH group. Nonetheless, previous studies showed promising results for the treatment of C1INH for several indications, among them transplant rejection, IR injury, burning injuries, and sepsis [34,35,36,37]. For example, Schelzig et al. investigated the effect of C1INH in an ex vivo hemoperfusion model of pig lungs with whole human blood. Ex vivo hemoperfusion was performed with fresh heparinized human blood plus/minus C1INH. Decreased hyperacute rejection was seen in the pig lungs treated with human blood plus C1INH compared to the control group. This effect was explained by the reduced C1q, C3, and C5b-9 activation in the C1INH group. In addition, a decrease in EC activation was seen, as lower concentrations of L- and P- selectin were demonstrated in the group treated with C1INH [37]. One important factor may be the dose dependence of C1INH actions. Dosages investigated in experimental studies often correspond to ten times the dose chosen for the PROTECT trial [38]. Interestingly, Buerke et al. confirmed a dose-dependence in the treatment with C1INH in a feline model of myocardial ischemia and reperfusion [39]. The administration of C1INH 10 min before reperfusion led to a decrease in 65% in myocardial necrosis. The largest effect was seen with a dose of 100 IU/kg [40,41]. In contrast, a dose of only 50 IU/kg was used in the current trial, which may not be sufficient to inhibit the activation of ECs. 

Our study has several limitations including the small sample size and the limited number of samples available. In our study, multiple comparisons were performed, so significant differences as described could be a chance result in the context of multiple statistical analyses. Conversely, it is possible that smaller differences are only detectable in a larger cohort of patients. The selected drug dosage of rhC1INH, may have been too low to influence EC activation. On the other hand, any influence may have been difficult to show as angiography was only a very weak trigger of acute renal injury and EC activation in this study population.

## 5. Conclusions

In the present post hoc analysis of a randomized controlled trial, EC activation was modest following CM administration during coronary angiography and not significantly increased by PCI. ChC1INH had no influence on EC activation. Future larger trials with more pronounced triggers and a higher dosage of rhC1INH are required to fully elucidate any association.

## Figures and Tables

**Table 1:** Baseline demographics, clinical and procedural characteristics, and laboratory parameters.

	All Patients n = 74	Placebo n = 37	rhC1INH n = 37
Age, years	76.7 ± 8.1	77 ± 9.3	76.3 ± 7
Male	52 (70.3)	27 (73)	25 (67.6)
**Comorbidities:**			
Diabetes mellitus	30 (40.5)	13 (35.0)	17 (46.0)
Coronary artery disease	42 (57.0)	19 (51.0)	23 (62.0)
Dyslipidemia	47 (63.5)	23 (62.2)	24 (65.0)
PAD	10 (13.5)	4 (11.0)	6 (16.0)
Smoking (previous and current)	44 (59.5)	21 (57.0)	23 (62.0)
CKD stage 3	62 (83.8)	31 (83.9)	31 (83.9)
CKD stage 4	11 (14.9)	6 (16.2)	5 (13.5)
CKD stage 5	1 (1.4)	0 (0)	1 (2.7)
**Medication:**			
Anticoagulation	28 (38.0)	16 (43.0)	12 (32.0)
Antiplatelet drugs	46 (62.0)	21 (57.0)	25 (67.6)
Statin	49 (66.0)	21 (57.0)	28 (76.0)
**Intervention:**			
PCI	28 (38.0)	14 (38.0)	14 (38.0)
**Laboratory parameters at baseline:**			
Creatinine, µmol/L	145.2 ± 66.88	139.39 ± 40.48	149.6 ± 85.36
eGFR, ml/min/1.73^2^	39.81 ± 9.62	39.84 ± 9.12	39.78 ± 10.22
Cystatin C, ng/mL	1.68 ± 0.50	1.68 ± 0.45	1.68 ± 0.59

Data are expressed in numbers (percentage) or mean ± SD. CKD = chronic kidney disease; PAD = peripheral artery disease, PCI = percutaneous coronary intervention; rhC1INH = recombinant human C1 inhibitor.

**Table 2:** Comparison of relative changes in serum concentrations of endothelial activation markers/CCL5 in patients according to the treatment with percutaneous coronary intervention (PCI) vs. coronary angiography only.

	Cystatin C Increase ≥ 10% within 24 h Compared to Baseline (n = 18)	No Cystatin C Increase ≥ 10% within 24 h Compared to Baseline (n = 53)	*p* Value
E- selectin baseline	31.0 ng/mL (19.4–49.6)	28.24 ng/mL (23.4–38.2)	*p* = 0.94
E-selectin 4 h	27.0 ng/mL (21.5–48.2)	28.4 ng/mL (22.7–38.6)	*p* = 0.849
E- selectin day 1	28.1 ng/mL (20.1–58.9)	30.0 ng/mL (23.5–44.4)	*p* = 0.98
ICAM-1 baseline	405.8 ng/mL (321.1–577.4)	411.0 ng/mL (354.8–488.0)	*p* = 0.90
ICAM-1 4 h	387.8 ng/mL (372.0–638.1)	393.6 ng/mL (342.2–471.1)	*p* = 0.43
ICAM-1 day 1	411.6 ng/mL (343.7–615.2)	416.4 ng/mL (370.6–510.4)	*p* = 0.69
VCAM-1 baseline	958.0 ng/mL (802.4–1284.6)	959.0 ng/mL (778.6–1148.3)	*p* = 0.80
VCAM-1 4 h	1164.7 ng/mL (723.0–1604.6)	996.0 ng/mL (834.0–1237.1)	*p* = 0.60
VCAM-1 day 1	1218.6 ng/mL (845.8–1451.7)	1084.4 ng/mL (854.4–1271.7)	*p* = 0.38
CCL5 baseline	17.9 ng/mL (14.6–68.3)	17.7 ng/mL (11.1–26.0)	*p* = 0.30
CCL5 4 h	23.2 ng/mL (13.6–46.7)	17.1 ng/mL (11.1–24.2)	*p* = 0.21
CCL5 day 1	19.5. ng/mL (13.2–48.0)	17.2 ng/mL (13.5–29.0)	*p* = 0.56

Median (25;75. Percentile).

## Data Availability

The datasets generated are available from the corresponding author on reasonable request (requiring prior ethics approval).

## Supplementary Materials

The following supporting information can be downloaded at: https://www.mdpi.com/article/10.3390/biomedicines12091956/s1, Figure S1: Relative change in % median serum concentration of E-selectin, ICAM-1, VCAM-1, and CCL5 according to the treatment with antiplatelet drugs; Table S1: Median serum concentrations (ng/mL) for endothelial activation markers/CCL5 in the overall study population during the first 48 h after coronary angiography; Table S2: Analysis of serum concentration of endothelial activation markers and CCL5 according to the treatment with PCI; Table S3: Analysis of serum concentrations of endothelial activation markers and CCL5 according to the treatment with antiplatelet drugs; Table S4: Analysis of serum concentrations of endothelial activation markers and CCL5 according to anticoagulation treatment; Table S5: Analysis of serum concentrations of endothelial activation markers and CCL5 according to treatment with rhC1INH/placebo.
