# Supplementary material for: Association of Endothelial Cell Activation with Acute Kidney Injury during Coronary Angiography and the Influence of Recombinant Human C1 Inhibitor—A Secondary Analysis of a Randomized, Placebo-Controlled, Double-Blind Trial
Source: Biomedicines. 2024 Aug 27;12(9):1956. doi: 10.3390/biomedicines12091956 (PMC11428207; doi:10.3390/biomedicines12091956)
Supplement: Supplementary file 1 [file biomedicines-12-01956-s001.zip › biomedicines-3146502-supplementary.pdf]

Supplementary Material

Supplementary Table S1: Median serum concentrations (ng/ml) for endothelial activation markers / CCL5 in the overall study population during the first 48 hours after coronary angiography

	Median [25; 75. percentile]
E-selectin baseline	27.45 [23.21; 38.17]
E-selectin 4 hours	27.92 [22.26; 38.59]
E-selectin day 1	29.67 [23.34; 44.39]
E-selectin day 2	29.80 [24.22; 44.40]
ICAM-1 baseline	411.00 [349.21; 495.25]
ICAM-1 4 hours	393.59 [346.63; 489.07];
ICAM-1 day 1	416.44 [370.56; 523.93]
ICAM-1 day 2	420.88 [366.31; 525.25]
VCAM-1 baseline	954.65 [782.79; 1140.66]
VCAM-1 4 hours	1005.15 [834.01; 1242.01]
VCAM-1 day 1	1107.17 [854.45; 1329.69]
VCAM-1 day 2	1052.87 [864.20; 1192.16]
CCL5 baseline	17.66 [11.49; 27.19]
CCL5 4 hours	18.25 [11.93; 24.81]
CCL5 day 1	17.39 [13.54; 29.21]
CCL5 day 2	32.17 [23.51; 44.95]

Supplementary Table S2: Analysis of serum concentration of endothelial activation markers and CCL5 according to the treatment with PCI

	**PCI yes= 28**	**PCI no= 46**	**Test**
**E-selectin baseline**	33.9 ng/ml (25.7; 40.7)	26.0 ng/ml (20.5; 36.4)	p = 0.054
**E-selectin 4 hours**	30.3 ng/ml (24.3; 40.3)	27.1 ng/ml (20.5; 36.8)	p = 0.212
**E-selectin day 1**	35.9 ng/ml (26.5; 53)	28.1 ng/ml (20.4; 39.8)	p = 0.038*
**E-selectin day 2**	34.7 ng/ml (26.9; 47.2)	26.5 ng/ml (21.2; 36.8)	p = 0.028*
**ICAM-1 baseline**	426.5 ng/ml (358.0; 509.3)	408.4 ng/ml (343.7; 478.5)	p = 0.504
**ICAM-1 4 hours**	378.9 ng/ml (329; 466.5)	404.8 ng/ml (363.5; 511)	p = 0.192
**ICAM-1 day 1**	430.4 ng/ml (381.8; 520.6)	412.5 ng/ml (349.3; 532.3)	p = 0.448
**ICAM-1 day 2**	420.4 ng/ml (350.5; 511.6)	422.1 ng/ml (374.8; 534.6)	p = 0.523
**VCAM-1 baseline**	922.3 ng/ml (780.2; 1129)	963.6 ng/ml (790.8; 1157.6)	p = 0.472
**VCAM-1 4 hours**	976.7 ng/ml (830.8; 1151.7)	1099.4 ng/ml (850.7; 1323.6)	p = 0.157
**VCAM-1 day 1**	1095.8 ng/ml (918.1; 1326.5)	1132.9 ng/ml (840.9; 1333.5)	p = 0.908
**VCAM-1 day 2**	952.2 ng/ml (859.8; 1128.0)	1088.7 ng/ml (864.2; 1246.6)	p = 0.289
**CCL5 baseline**	19.6 ng/ml (14.7; 28.8)	15 ng/ml (10.5; 26.5)	p = 0.204
**CCL5 4 hours**	20.2 ng/ml (9.8; 25.5)	17.5 ng/ml (12.0; 24.4)	p = 0.902
**CCL5 day 1**	17.8 ng/ml (13.5; 29.0)	16.9 ng/ml (13.7; 30.3)	p = 0.773
**CCL5 day 2**	34.1 ng/ml (26.9; 47.2)	26.5 ng/ml (21.2; 36.8)	p = 0.828

* Note: Not significant after Bonferroni correction for multiple comparisons;

Median (25;75. Percentil)

Supplementary Table S3: Analysis of serum concentrations of endothelial activation markers and CCL5 according to the treatment with antiplatelet drugs

	**Antiplatelet drugs yes n= 46**	**Antiplatelet drugs no= 28**	**Test**
**E-selectin baseline**	28.2 ng/ml (20.7; 35.0)	27.1 ng/ml (20.7; 35.0)	p= 0.465
**E-selectin 4 hours**	28.4 ng/ml (21.6; 39.4)	27.1 ng/ml (23.9; 32.4)	p= 0.597
**E-selectin day 1**	31.6 ng/ml (24.1; 44.4)	28.0 ng/ml (20.8; 46)	p=0.501
**E-selectin day 2**	30.0 ng/ml (25.1; 46)	27.5 ng/ml (21.0; 38.7)	p=0.497
**ICAM-1 baseline**	412.3 ng/ml (330.2; 525.1)	408.4 ng/ml (353.6; 469.2)	p=0.734
**ICAM-1 4 hours**	384.3 ng/ml (332.0; 474.3)	416.8 ng/ml (378.9; 501.1)	p=0.137
**ICAM-1 day 1**	418.0 ng/ml (354.8; 5.0)	414.0 ng/ml (373.5; 550.9)	p=0.719
**ICAM-1 day 2**	420.4 ng/ml (351.2-523.4)	422.1 ng/ml (382.0; 545.3	p=0.430
**VCAM-1 baseline**	944.9 ng/ml (778.6; 1160.7)	964.4 ng/ml (813.3; 1094.6)	p=0.898
**VCAM- 1 4 hours**	991.9 ng/ml (834.0; 1209.5)	1070.9 ng/ml (824.7; 1403.0)	p=0.341
**VCAM-1 day 1**	1078.6 ng/ml (836.4; 1319.7)	1146.4 ng/ml (905.1; 1376.5)	p=0.437
**VCAM -1 day 2**	973.6 ng/ml (862.7; 1175.9)	1095.101 ng/ml (864.2; 1468.5)	p=0.225
**CCL5 baseline**	**19.2 ng/ml (13.9; 29)**	**13.5 ng/ml (8.4; 19.4)**	**p=0.020***
**CCL5 4 hours**	**20.2 ng/ml (14; 28.8)**	**14.2 ng/ml (9.2; 21)**	**p=0.032***
**CCL5 day 1**	19.5 ng/ml (14.6; 32.3)	15.9 ng/ml (10.5; 27.8)	p=0.137
**CCL5 day 2**	34.1 ng/ml (24.2; 51.9)	28.5 ng/ml (17.9; 40.5)	p=0.175

* Note: Not significant after Bonferroni correction for multiple comparisons

Median (25;75. Percentil)

Supplementary Table S4: Analysis of serum concentrations of endothelial activation markers and CCL5 according to anticoagulation treatment

	**Anticoagulation yes= 28**	**Anticoagulation no= 46**	**Test**
**E-selectin baseline**	25.8 ng/ml (20.5-32.5)	32.7 ng/ml (24.0-43.7)	p = 0.023*
**E-selectin 4 hours**	26.7 ng/ml (19.2-31.0)	30.4 ng/ml (23.7-43.0)	p = 0.042*
**E-selectin day 1**	28.1 ng/ml (20.3-38.0)	34.3 ng/ml (25.6-51.3)	p = 0.085
**E-selectin day 2**	27.5 ng/ml (21.5-38.4)	30.7 ng/ml (25.0-46.7)	p = 0.349
**ICAM-1 baseline**	416.6 ng/ml (350.9-469.2)	405.8 ng/ml (343.6-542.2)	p = 0.734
**ICAM-1 4 hours**	433.1 ng/ml (376.6-485.9)	384.5 ng/ml (331.9-490.7)	p = 0.240
**ICAM-1 day 1**	420.9 ng/ml (368.1-517.9)	412.5 ng/ml (370.6-523.9	p = 0.953
**ICAM-1 day 2**	456.0 ng/ml (377.4-548.9	409.6 ng/ml (354.5-521.5	p = 0.266
**VCAM-1 baseline**	1068.0 ng/ml (818.2-1171.4)	931.8 ng/ml (777.5-1136.3)	p = 0.198
**VCAM- 1 4 hours**	1061.9 ng/ml (961.8-1355.4)	991.9 ng/ml (792.5-1208.5)	p = 0.137
**VCAM -1 day 1**	1151.0 ng/ml (986.0-1376.5)	1054.3 ng/ml (835.9-1271.7)	p = 0.180
**VCAM- 1 day 2**	**1132.0 ng/ml (1035.1-1427.8)**	**949.1 ng/ml (842.2-1115.0)**	**p = 0.005**
**CCL5 baseline**	13.7 ng/ml (8.9-21.3)	18.7 ng/ml (13.3-29.3)	p = 0.029*
**CCL5 4 hours**	15.2 ng/ml (9.0-23.0)	19.5 ng/ml (13.2-26.5)	p = 0.055
**CCL5 day 1**	16.3 ng/ml (12.7- 20.2)	19.6 ng/ml (14.1-39.4)	p = 0.042*
**CCL5 day 2**	28.5 ng/ml (20.0-39.9)	34.1 ng/ml (24.5-53.8)	p = 0.123

* Note: Not significant after Bonferroni correction for multiple comparisons

Median (25;75. Percentil)

Supplementary Table S5: Analysis of serum concentrations of endothelial activation markers and CCL5 according to treatment with rhC1INH/placebo

	**Placebo (n = 37)**	**rhC1INH (n = 37)**	**Test**
**E-selectin baseline**	27.8 ng/ml (25.1-40.5)	27.0 ng/ml (20.5-37.3)	p = 0.323
**E-selectin 4 hours**	27.2 ng/ml (24.0-41.0)	28.3 ng/ml (18.9-35.5)	p = 0.556
**E-selectin day 1**	30.3 ng/ml (25.4-49.2)	28.7 ng/ml (21.5-41.7)	p = 0.429
**E-selectin day 2**	30.3 ng/ml (24.3-43.4)	29.1 ng/ml (24.1-46.0)	p = 0.968
**ICAM-1 baseline**	429.5 ng/ml (354.0-540.3)	396.1 ng/ml (339.0 -465.7)	p = 0.132
**ICAM-1 4 hours**	395.8 ng/ml (371.2-496.3)	391.4 ng/ml (326.8-479.0)	p = 0.479
**ICAM-1 day 1**	413.4 ng/ml (379.6-524.4)	417.2 ng/ml (366.3-528.1)	p = 0.880
**ICAM-1 day 2**	419.9 ng/ml (361.8-516.7)	424.5 ng/ml (370.0-543.0)	p = 0.607
**VCAM-1 baseline**	963.1 ng/ml (786.5-1132.1)	954.4 ng/ml (781.5-1163.0)	p = 0.795
**VCAM-1 4 hours**	993.1 ng/ml (852.5-1222.4)	1075.4 ng/ml (832.4- 1281.6)	p = 0.630
**VCAM-1 day 1**	1078.7 ng/ml (827.9-1287.2)	1115.6 ng/ml (918.1-1334.0)	p = 0.429
**VCAM-1 day 2**	967.5 ng/m (967.5-1146.4)	1084.6 ng/ml (905.7-1280.3)	p = 0.099
**CCL5 baseline**	17.6 ng/ml (10.9-26.0)	18.6 mg/ml (11.4-30.1)	p = 0.589
**CCL5 4 hours**	16.9 ng/ml (8.9-26.1)	18.3 ng/ml (13.7-24.6)	p = 0.452
**CCL5 day 1**	16.6 ng/ml (12.8-28.4)	19.5 ng/ml (14.7-32.2)	p = 0.166
**CCL5 day 2**	29.2 ng/ml (20.7-47.5)	33.0 ng/ml (23.7-44.9)	p = 0.561

Median (25;75. Percentil)

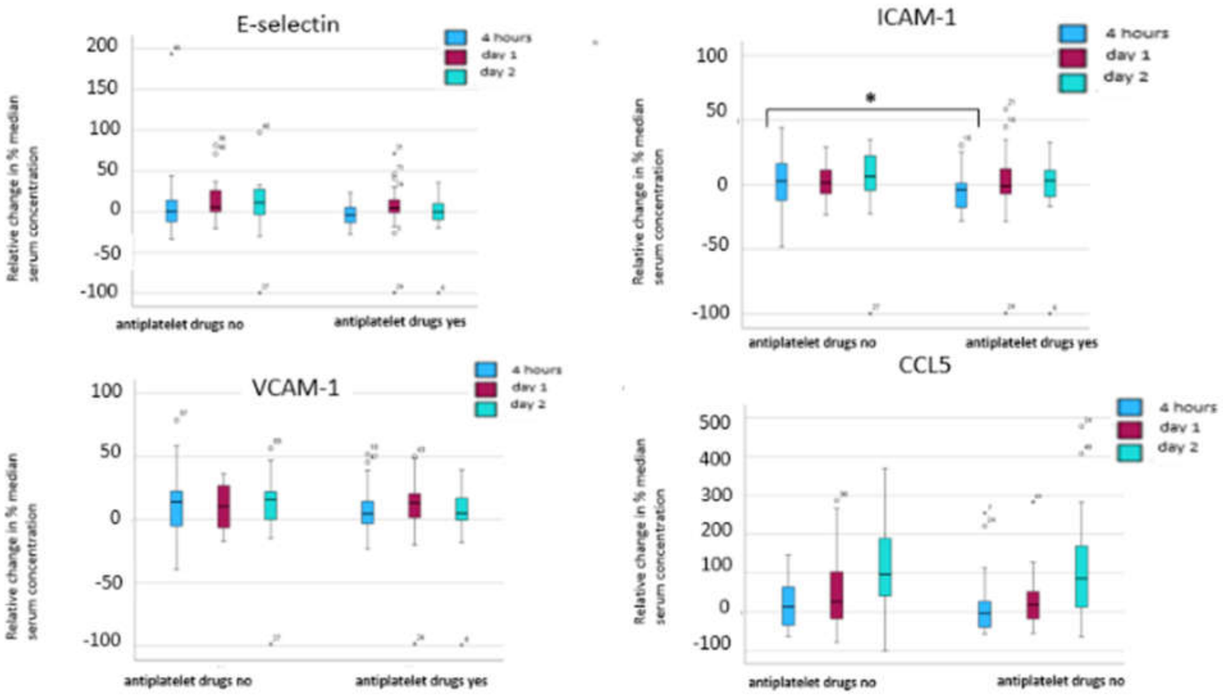

Supplementary Figure S1: Relative change in % median serum concentration of E-selectin, ICAM-1, VCAM-1 and CCL5 according to the treatment with antiplatelet drugs.
